# Structural Characterization of Outer Membrane Components of the Type IV Pili System in Pathogenic Neisseria

**DOI:** 10.1371/journal.pone.0016624

**Published:** 2011-01-31

**Authors:** Samta Jain, Katarzyna B. Mościcka, Martine P. Bos, Emilia Pachulec, Marc C. A. Stuart, Wilko Keegstra, Egbert J. Boekema, Chris van der Does

**Affiliations:** 1 Department of Molecular Microbiology, Groningen Biomolecular Sciences and Biotechnology Institute and the Zernike Institute for Advanced Materials, University of Groningen, Haren, The Netherlands; 2 Department of Biophysical Chemistry, Groningen Biomolecular Sciences and Biotechnology Institute, University of Groningen, Groningen, The Netherlands; 3 Department of Molecular Microbiology, Faculty of Science, Utrecht University, Utrecht, The Netherlands; 4 Department of Ecophysiology, Max-Planck-Institute for Terrestrial Microbiology, Marburg, Germany; University of Hyderabad, India

## Abstract

Structures of the type IV pili secretin complexes from *Neisseria gonorrhoeae* and *Neisseria meningitidis,* embedded in outer membranes were investigated by transmission electron microscopy. Single particle averaging revealed additional domains not observed previously. Secretin complexes of *N. gonorrhoeae* showed a double ring structure with a 14–15-fold symmetry in the central ring, and a 14-fold symmetry of the peripheral ring with 7 spikes protruding. In secretin complexes of *N. meningitidis*, the spikes were absent and the peripheral ring was partly or completely lacking. When present, it had a 19-fold symmetry. The structures of the complexes in several *pil* mutants were determined. Structures obtained from the *pilC1/C2* adhesin and the *pilW* minor pilin deletion strains were similar to wild-type, whereas deletion of the homologue of *N. meningitidis* PilW resulted in the absence of secretin structures. Remarkably, the *pilE* pilin subunit and *pilP* lipoprotein deletion mutants showed a change in the symmetry of the peripheral ring from 14 to 19 and loss of spikes. The *pilF* ATPase mutant also lost the spikes, but maintained 14-fold symmetry. These results show that secretin complexes contain previously unidentified large and flexible extra domains with a probable role in stabilization or assembly of type IV pili.

## Introduction

Neisseria species are Gram-negative β-proteobacteria, whose pathogenic members *Neisseria meningitidis*, which normally inhabits the human nasopharynx, and *Neisseria gonorrhoeae*, which normally colonizes urogenital mucosal surfaces, are responsible for bacterial meningitis and septicemia, and the sexually transmitted disease gonorrhea, respectively. During the infection process, several factors contribute to the interaction with the host cells [1]. Among these factors are type IV pili which mediate binding of the bacteria to the host cells. Type IV pili are long fibrous structures extending from the bacterial surface which can be extended and retracted [2], [3]. They are involved in a variety of processes; not only do they mediate cellular attachment to tissue receptors [1], [4], but they are also involved in several other processes, including bacterial auto-agglutination [5], [6], twitching motility [7], biofilm formation [3], [8], [9], [10], and natural competence for DNA uptake [11], [12], [13].

Type IV pili are dynamic structures which consist of approximately 500–2000 subunits of the major pilin protein, PilE [14] and which are assembled and disassembled by a complex machinery of approximately 10 conserved core proteins and several additional proteins [2], [3], [15]. This machinery shows similarity to the complexes involved in secretion of proteins via the type II secretion pathway [16], [17]. The nomenclature of components of type IV pili systems often differs between organisms. In this manuscript we will refer to the *N. gonorrhoeae* genes and proteins if not indicated otherwise. The first step of pilus assembly is the insertion of the pilin into the cytoplasmic membrane. After membrane insertion, the leader peptide is both cleaved at the cytosolic side of the membrane and methylated on the N-terminal amino acid by the pre-pilin peptidase PilD [18], [19]. The PilE subunits are assembled and extruded from the inner membrane by the PilF hexameric ATPase (a homologue of GspE, and a member of the AAA chaperone/mechanico-enzyme family) with the aid of a polytopic inner membrane protein, PilG [20]. Remarkably, PilT which is a similar ATPase to PilF, is involved in the disassembly of the PilE subunits at the cytoplasmic membrane. Disassembly takes place at a rate of approximately 700 pilin subunits/s, resulting in retraction of the pilus with a force of over 100 pN [21], [22]. Several other proteins, called pseudo-pilins or minor pilins, are similarly processed by PilD and can also be integrated into the growing pilus, and were proposed to affect pilus dynamics by influencing the membrane-localization and/or polymerization state [23]. The pilus passes the outer membrane through PilQ [24], [25]. PilQ is one of the most abundant Neisserial outer membrane proteins and it has previously been estimated that PilQ comprises 10–13% of the total outer membrane proteins [26]. PilQ is a member of the GspD secretin superfamily of integral outer membrane proteins involved in type IV pili and in type II and type III secretion systems [27].

Transmission electron microscopy (TEM) of purified members of the secretin superfamily, such as XcpQ and PilQ from *Pseudomonas aeruginosa* [28], PulD from *Klebsiella oxytoca* [29], [30], [31], the pIV filamentous phage protein [32], GspD from *Vibrio cholerae* [33] and PilQ of *N. meningitidis* [24], [25], [34], [35], indicated that these proteins form a multimeric ring-like structure. A 3D structure of the *N. meningitidis* PilQ (Nme PilQ) was determined by using single particle averaging methods applied to transmission electron microscopy (EM) images of the purified multimer visualized by cryo-negative EM staining. This structure showed 4-fold rotational symmetry (and 12 fold quasi-symmetry) with four ‘arm’-like structures extending from the structure and a large central cavity which was closed on both sides. [24], [25], [34], [35]. The observed structure was flexible, and showed conformational changes upon interaction with isolated pili [25] and purified NMe PilP [36]. A higher resolution 3D structure of a secretin was obtained for *K. oxytoca* PulD [29], [30], [31]. This complex consists of a dodecameric structure composed of a closed disc with ring-like structures on both sides. The two rings form chambers on either side of a central plug that is part of the middle disc. A recently published dodecameric cryo-EM structure of the purified GspD secretin of *Vibrio cholera* shows a 200 Å long complex with a periplasmic domain, an outer membrane domain and a unique extracellular cap. The structure was obtained in its “closed” state and has an outer diameter of 155 Å. It has a prominent periplasmic gate and a conserved constricted region. It was proposed that this region interacts with the substrate and renders conformational changes to the structure for toxin secretion [33].

Members of the secretin superfamily often interact with small lipoproteins, also known as pilotins, or pilot proteins. These lipoproteins are involved in oligomerization, stabilization, and/or outer membrane localization of the secretin. For example, the *K. oxytoca* PulD requires the PulS pilotin for proper outer membrane association; in its absence, PulD remains associated with the inner membrane [37]. Similarly the *Shigella flexneri* MxiM and *Yersinia enterocolitica* YscW pilotins are required for outer membrane localization of the Type III secretion secretins MxiD and YscC, respectively [38], [39]. The interaction between MxiM and MxiD has been studied using NMR spectroscopy [40]. It has been demonstrated that Nme PilP and Nme PilW interact with Nme PilQ. Purified Nme PilP was shown to interact with Nme PilQ and was proposed to localize to the cap region of the Nme PilQ structure [36]. Although Nme PilQ does not need Nme PilP for its stabilization or membrane localization, Nme *pilP* mutants showed a loss of piliation and of natural competence [36]. In a Nme *pilW* deletion mutant, the total amount of outer membrane localization of Nme PilQ monomers was not changed, but the stability of the Nme PilQ multimer was strongly affected [41]. Another protein that has been proposed to interact with PilQ is PilC. Two copies of *pilC* (*pilC1* and *pilC2*) are found in pathogenic Neisseria species. In *N. gonorrhoeae*, each copy can function as a pilus tip adhesin, while in *N. meningitidis*, only PilC1 promotes adhesion [42]. The Nme PilC proteins are associated with the outer membrane but can also be recovered from purified pili, where they seem to be located at the top of the pilus [43].

Although the observations made by different authors have been useful in establishing that secretins adopt ring-like structures with 12–14 fold symmetry, there are still many remaining questions. All structural information about the secretins obtained to date has been obtained from purified proteins, and structural information about the interaction of the secretins with other components is lacking. Multi-subunit membrane complexes can loose subunits during the purification procedure [44]. Therefore we set out to study the structure of the PilQ secretin within the membrane using Transmission Electron Microscopy and single particle averaging. To obtain further information on the PilQ complex, we studied the structure of the complex in membranes derived from different *pil* deletion mutants. Implications for the assembly and structure of the observed PilQ mega-complex are discussed.

## Results

### Transmission electron microscopy on isolated membranes and whole cells of *Neisseria gonorrhoeae*

**Figure 1:**
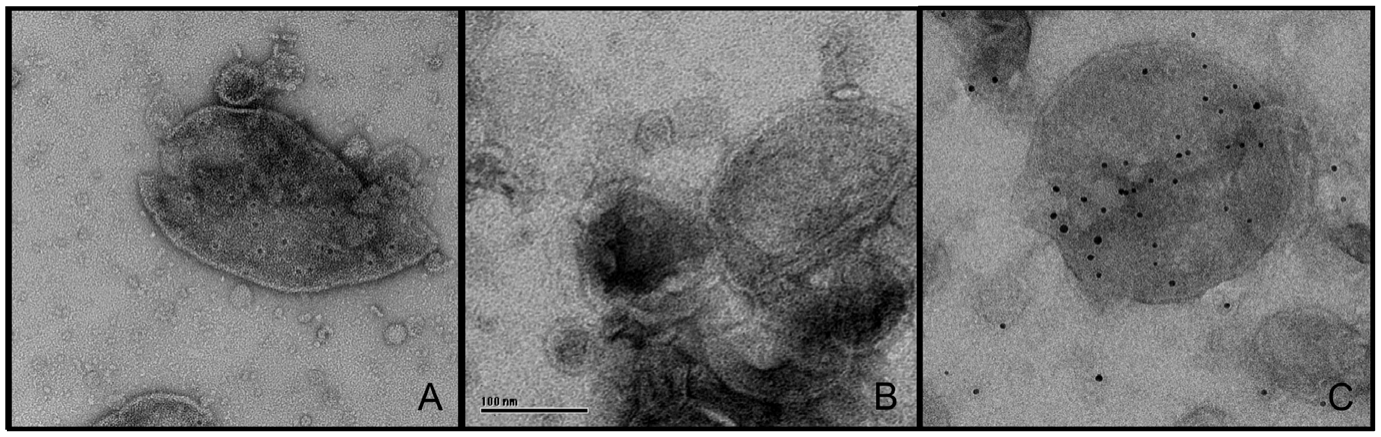
Overview of negatively stained *N. gonorrhoeae* membranes. (A) Membranes isolated from strain MS11. (B) Membranes isolated from a *pilQ* deletion mutant strain. (C) Immungold labeling of membranes from strain MS11 with PilQ antibody. The scale bar is 1000 Å.

To study the structure of the PilQ secretin of *N. gonorrhoeae* in its native environment, total membranes were isolated and separated on a sucrose gradient. Fractions containing the highest amount of PilQ (from 45 to 51% (w/v) sucrose) were collected and concentrated. This fraction contained both inner and outer membranes as determined by antibodies against SecY and DsbA inner membrane markers) and Omp85 and Imp (outer membrane markers). Although several methods, including different disruption methods in combination with a large variety of density gradients were tested to separate inner and outer membranes, no complete separation was obtained. PilQ containing fractions were analyzed with transmission electron microscopy. Both inner membranes, which appear as vesicles, and outer membranes, which appear as flattened sheets, could be identified [45]. Roughly 25% of the vesicles seem to be derived from inner membranes. The membranes form intact closed vesicles because upon air-dried negative staining the membranes collapse and become superimposed. This can be seen at the edges where a white rim marks the curvature (Figure 1A). The outer membranes contained prominent stain-filled indentations (Figure 1A) which were absent in the inner membranes, with an average density of 350 indentations per µm^2^

**Figure 2:**
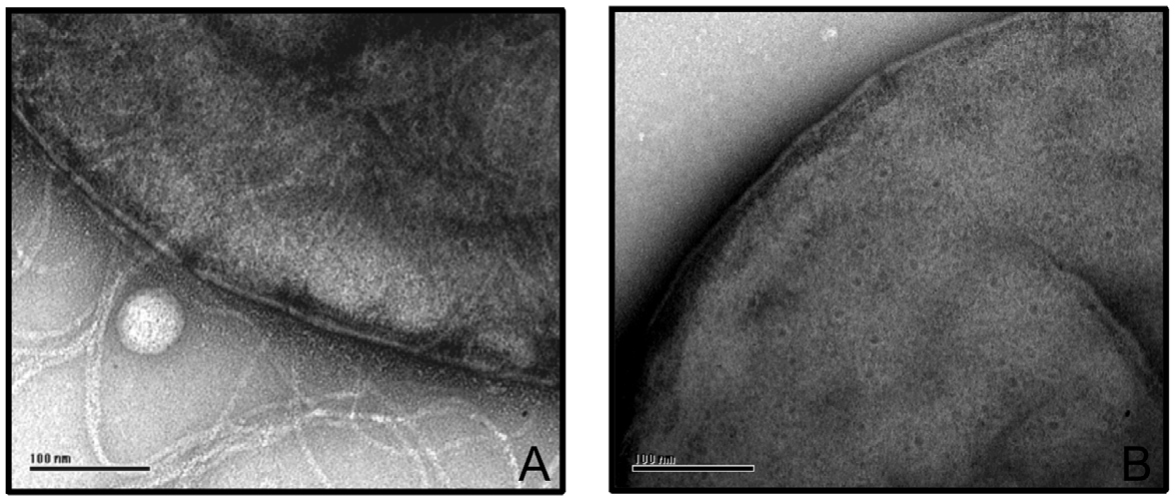
Secretin structures observed by negative stain imaging of whole cells. (A) Piliated and (B) non-piliated cells of *N. gonorrhoeae* strain were imaged by electron microscopy. The scale bar is 1000 Å.

Since these stain-filled indentations were most likely formed by the PilQ secretin, a *pilQ* deletion strain was constructed. Comparison of the outer membrane enriched samples of MS11 and the *pilQ* deletion strain confirmed both the abundance of PilQ in the outer membrane samples, and the absence of PilQ in the deletion strain (see Figure S1A). Isolated membranes of the *pilQ* deletion strain were analyzed by electron microscopy (Figure 1B). The stain filled indentations were absent in the membranes of the *pilQ* deletion strain demonstrating that they are indeed related to the presence of PilQ. Interestingly, the stain filled indentations were also evident on whole cells of *Neisseria gonorrhoeae* when observed using electron microscopy (Figure 2). While comparing piliated and non piliated cells, some of the thin, long type IV pili structures on the piliated cells seemed to extend from the stain filled indentations. These results further demonstrated the outer membrane localization of the stain filled indentations (Figure 2A and 2B). To further confirm that these indentations contain PilQ, nanogold labeling was performed on the outer membrane enriched fractions using a *N.meningitidis* PilQ monoclonal antibody [46]. This monoclonal antibody specifically cross reacts with *N.gonorrhoeae* PilQ (see Figure S1B). Here we observed labeling of the indentations in PilQ containing membranes (See Figure 1C). When similar experiments were performed in the absence of the PilQ antibody only very low levels of nano-gold labeling were observed (see Figure S3A). Similar very low levels of nano-gold labeling were observed for inner membranes and for membranes derived from the *pilQ* deletion strain (Figure S3B). To-gether this showed that labeling is specific for the presence of PilQ in the indentations. Since the gold labeling influences the alignment procedure, single particle averaging was not performed on gold labeled particles instead, about a hundred images were visually analyzed (Figure 1C, see also Figure S2). Labeling was strongly reduced for the observed inner membranes, or when control experiments were performed in the absence of the PilQ antibody (Figure 1C).

### Projection structure of the *N. gonorrhoeae* PilQ complex

**Figure 3:**
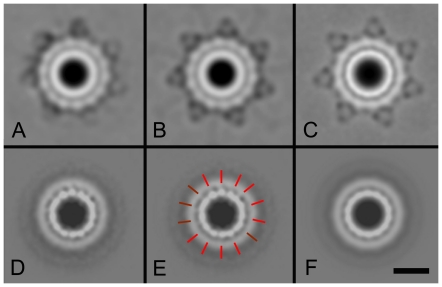
Class averages of single particle electron microscopy images of the PilQ complex from *N. gonorrhoeae*. (A) wild-type projection map (B) with 7-fold symmetry imposed on the peripheral spikes (C) with 7-fold symmetry imposed using the class average of (B) as template (D) class average of the central ring, after masking out the second ring and spikes (E) central ring 2D map with positions of densities indicated (F) central ring 2D map with imposed 14-fold symmetry. The projection map of the PilQ complex in frame A has a resolution of 20 Å. The scale bar is 100 Å.

To further analyze this PilQ-containing structure, a large data set of about 20,000 single projections of the stain-filled indentations was obtained from EM images and analyzed by single particle analysis. After several cycles of multi-reference alignment, multivariate statistical analysis and classification, final class sums from all analyzed particles were obtained (Figure 3A). The 2D map shows a circular particle composed of a double ring with extending spike-like densities. The central ring has a diameter of 150 Å and has a large central cavity, whereas the peripheral ring has a diameter of 210 Å. The spikes further extend the diameter to 310 Å. The second ring has a 14-fold symmetry, while the spike-like densities show a 7-fold symmetry. After applying 7-fold symmetry, the features of both the second ring and the spikes improve (Figure 3B). When this figure was used for further improvement as a reference in a next alignment procedure, it appears that the spike features became stronger, but at the cost of the resolution of the peripheral ring, which now becomes less well defined (Figure 3C). This suggests that the structure has some flexibility between the second ring and the spikes. The symmetry of the central ring could not be resolved from either the class averages without symmetry applied or from the class averages with 7-fold symmetry applied. In an attempt to determine the symmetry of the central ring, the second ring was masked out during analysis. After repeated alignment and classification, the final projection map showed two striking features. First, densities in the central ring come into focus (Figure 3D). At least 11 densities are well separated, with a average center-to-center distance of about 25 Å (red bars, Figure 3E). However, in two areas the features are not well resolved (blurred red bars), despite the fact that we increased the number of analyzed projections from 20,000 to 36,000. This indicates that at the current resolution of this map, which is in the range of the 25 Å of the center-to-center distance of the central ring densities, we cannot prove the symmetry. However, it appears most likely that the symmetry is 14 or 15. By imposing 14-fold symmetry, as performed in Figure 3F, the features become stronger as compared with any other imposed symmetry between 12 and 16. A second result from this analysis is the total disappearance of the features of the second ring. This can either be caused by a symmetry mismatch between the central and peripheral rings or by a flexible association of the central and peripheral rings.

### Transmission electron microscopy on isolated membranes of *N. meningitides*

**Figure 4:**
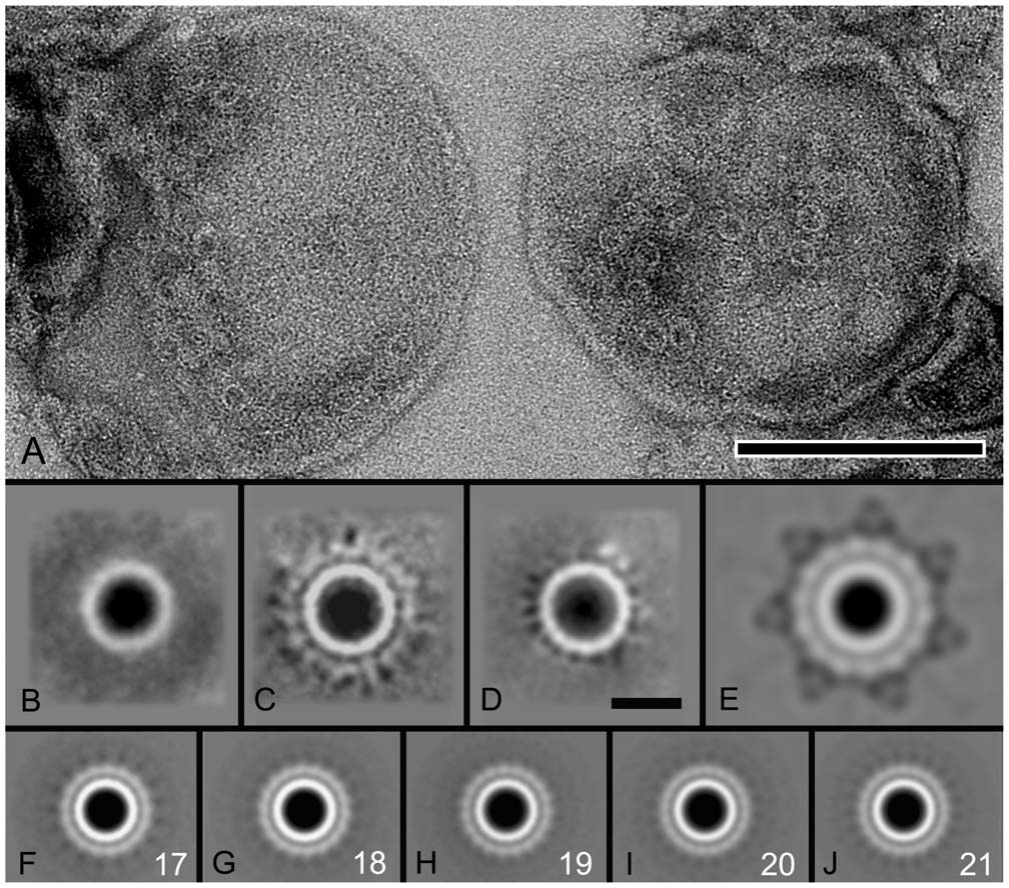
Electron microscopy analysis of the PilQ complexes in isolated membranes. (A) *N. meningitidis* membranes negatively stained with 2% uranyl acetate. (B–D) Average of 8,000 projections of the PilQ complexes from *N. meningitidis* showing classes with single, double and incomplete rings, respectively. (E) Average of 10,000 projections of PilQ complex from *N. gonorrhoeae* showing central and the peripheral ring having 14-fold symmetry. (F-J) To investigate the number of copies of the peripheral ring, 17- to 21-fold rotational symmetry (white numbering) was imposed on the classes after completion of the analysis. The strongest rotational symmetry within the second ring is in frame H. The scale bar for frame (A) is 1000 Å, and equals 100 Å for frame (B–J).

To enable us to compare the previously published structure of the purified Nme PilQ complex with the structure observed in the membrane, membranes of *N. meningitidis* were isolated and analyzed by transmission electron microscopy. The membranes of *N. meningitidis* also showed the presence of indentations, but in much smaller numbers compared to *N. gonorrhoeae*. Moreover, the pores were less homogeneously distributed in the membrane, and some of them are found in small clusters (Figure 4A). Single particle analysis showed a structure composed of only one ring (Figure 4B) or with an additional second ring in about 20% of the data set (Figure 4C). Some particles (10%) showed an incomplete second ring (Figure 4D). The central and peripheral rings have the same diameter as observed in the *N. gonorrhoeae* structure (Figure 4E). Remarkably, there was no indication of spikes attached to the second ring. Comparison with the previously published purified Nme PilQ structure, which has a diameter of approximately 15.5 to 16.5 nm and a 6.0- to 6.5-nm-diameter cavity showed that PilQ forms the central ring of both the *N. meningitidis* and *N. gonorrhoeae* structures, whereas the peripheral ring and the spikes are formed by additional proteins. Remarkably, the symmetry number of the peripheral ring observed for *N. meningitidis* was substantially bigger than that observed for *N. gonorrhoeae*, indicating that this ring is possibly composed of a larger number of copies of the same or a smaller protein. Symmetry analysis performed to evaluate the copy number shows that imposing 2-, 3-, or 7- fold does not enhance this feature. Since the motif is smaller than in *N. gonorrhoeae*, symmetries above 14 were evaluated (Figure 4F–J). This approach strongly pointed to a 19-fold symmetry in the second ring. Based on the high similarities of the PilQ proteins of *N. meningitidis* and *N. gonorrhoeae* (89% identity and 91% similarity), the observed differences were unexpected. To ensure that the observed differences in the PilQ complexes in the membranes of the *N. gonorrhoeae* MS11 and the *N. meningitidis* HB-1 strains are species specific, membranes of different strains were isolated. Two additional *N. meningitidis* strains, strain H44/76, the wild-type parent of HB-1 (to ensure that the absence of the capsule locus did not affect PilQ appearance), and wild-type strain M986, belonging to a different clonal lineage were tested. Furthermore, *N. gonorrhoeae* strain FA1090 was examined. Both *N. meningitidis* strains gave very similar results as strain HB-1. Similarly FA1090 gave identical results as were obtained for MS11. To further exclude that the strains used in our study contained any mutations in *pilP* or *pilQ*, the entire *pilP/Q* operon and flanking regions were sequenced, but no differences were observed with the published sequences [47]. One of the differences between the *N. meningitidis and N. gonorrhoeae* PilQ is the presence of a small octapeptide (PAKQQAAA) basic repeat of which four to seven copies are present in *N. meningitidis* PilQ, whereas *N. gonorrhoeae* PilQ contains either two or three copies [48]. However, the 14-fold symmetry of the peripheral ring and the spikes were still observed in the *N. gonorrhoeae* strain in which the 2 repeats of MS11 were replaced with the 6 repeats of *N. meningitidis* strain HB1 (data not shown).

### Structure and assembly of the PilQ complex of *N. gonorrhoeae*

**Figure 5:**
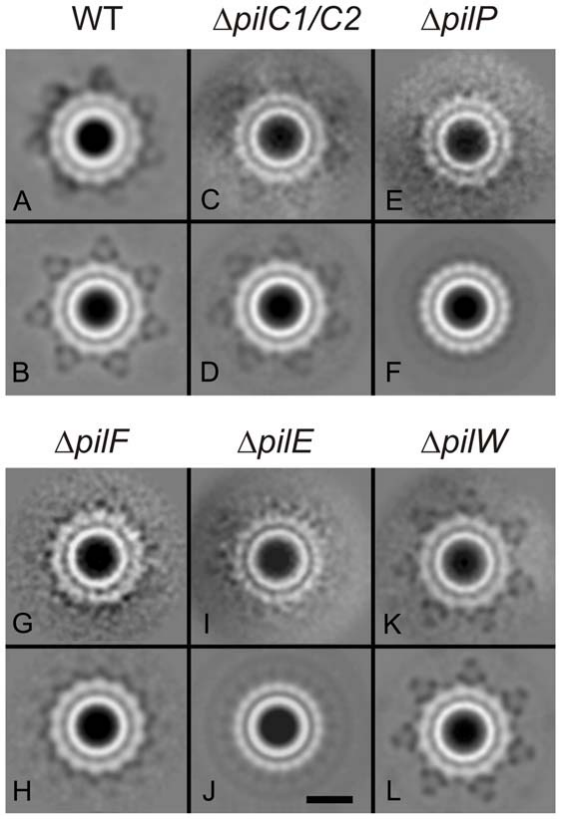
Single particle analysis of particles derived from membranes of different *N. gonorrhoeae* deletion mutant strains. Projection maps derived from membranes isolated from (A) *N. gonorrhoeae* strain MS11, (B) with 7-fold symmetry imposed, (C) the *pilC1/C2* deletion mutant, (D) the *pilC1/C2* deletion mutant with 7-fold symmetry imposed, (E) the *pilP* deletion mutant, (F) the *pilP* deletion mutant with 19-fold symmetry imposed, (G) the *pilF* deletion mutant, (H) the *pilF* deletion mutant with 7-fold symmetry imposed, (I) the the *pilE* deletion mutant, (J) the *pilE* deletion mutant with 19-fold symmetry imposed, (K) the *pilW* deletion mutant, and (L) the *pilW* deletion mutant with 7-fold symmetry imposed, Scale bar equals 100 Å.

Our analysis of the structure of the PilQ complex in isolated outer membranes unequivocally shows extra domains, i.e. a peripheral ring with associated spikes, not observed in purified Nme PilQ complexes. To attempt to identify these novel features, we set out to generate deletion mutants for genes of possible candidates for the extra observed densities. PilC is a protein normally present in two copies, is involved in adhesion to epithelial cells, and is located in the outer membrane and at the tip of the pilus. Since the *N. gonorrhoeae* MS11 strain used for our study, contains a non functional copy of *pilC1* that is not expressed due to a frame-shift mutation [49], we generated a *pilC2* deletion mutant in MS11. The *pilC1/C2* mutation resulted in non-piliated cells, as seen in previous studies. The *pilC1* gene was sequenced in the *pilC2* deletion mutant and this re-confirmed the presence of the frame-shift mutation. Hence, we conclude that a true *pilC1/C2* mutant was generated. Single particle analysis was performed and 6,000 projections were analyzed. The *pilC1/C2* mutant yielded projection maps showing a similar structure as observed for wild-type with the presence of central and peripheral rings and 7 extending spikes (Figure 5C). Again, these features became more visible after imposing symmetry (Figure 5D). This demonstrated that PilC is not a subunit of the observed PilQ complex.

In a next step, we generated a deletion mutant of *N. gonorrhoeae* NgonM_03101, a small (28 kDa) lipoprotein containing six tetratricopeptide repeats (TPR) motifs. *N. gonorrhoeae* NgonM_03101 is a homologue of Nme PilW and *Pseudomonas aeruginosa* PilF, which have been shown to be involved in stabilization of the PilQ oligomer [41], [50], [51]. In membranes isolated from the *NgonM_03101* deletion mutant, no stain filled indentations were observed demonstrating that NgonM_03101 is involved either in assembly or stabilization of the PilQ oligomer. Since no structures were observed, we cannot discriminate between the possibility that NgonM_03101 functions as a chaperone for oligomerisation of the secretin, or that it is part of the larger PilQ complex.

PilP is an 18 kDa lipoprotein shown to interact with PilQ. Both in *N. meningitidis* and *N. gonorrhoeae pilP* and *pilQ* are co-transcribed [36]. *pilP* mutants show a loss of piliation and natural competence [52]. In a previous study additional densities were observed when purified Nme PilQ was incubated with recombinant Nme PilP [36]. To study the localization of PilP in PilQ-containing complexes within the membrane, a *pilP* deletion mutant was created. Western blotting confirmed the expression of PilQ in the *pilP* deletion mutant strain. An increased degradation of full length PilQ was however observed (Figure S4) and the observed density of indentations in the membranes derived from the *pilP* deletion mutant was reduced to ∼30% compared to the wild-type membranes, suggesting that PilP influences the stability of PilQ multimers. Differences were observed when the class averages of 6,000 particles of the pilP mutant without (Figure 5E) and with applied symmetry (Figure 5F) were compared to the class averages obtained from wild-type membranes (Figure 5A and B). The *pilP* deletion mutant not only lost the extending spike-like densities, but remarkably the symmetry of the peripheral ring changed from 14 to 19. Even more surprisingly, the structure of the PilQ-containing complex in membranes derived of the *N. gonorrhoeae pilP* deletion mutant showed a notable similarity to the structure of the PilQ complex in *N. meningitidis* membranes. Based on a possible localization of PilP between the central and peripheral rings of the PilQ complex, it could be proposed that absence of PilP affects the interface between the central and peripheral ring, resulting in a reassembly of the second ring. The reassembled second ring does not appear to be able to bind the spike-like extensions.

It has been previously demonstrated that incubation of purified Nme PilQ complexes with isolated pili can induce structural changes in Nme PilQ [25]. To compare the structures of PilQ-containing complexes that have interacted with the pilus and those that have not interacted with the pilus, membranes derived from *pilF* and *pilE* deletion mutants were studied. PilF is an ATPase, localized in the inner membrane and essential for the assembly and extrusion of pilin subunits. PilE is the pilus subunit, which forms thin pilin filaments of ∼60–80 Å [53]. When the class averages of 6,000 particles obtained from membranes of the *pilF* and *pilE* deletion mutants without (Figure 5G and 5I) and with applied symmetry (Figure 5H and 5J) were compared to the class averages obtained from wild-type membranes, it appeared that the secretin structures of both mutants lost the extending spike-like densities. This suggests that the secretin complex changes its conformation upon interaction with the pilus resulting in assembly of the extending spike-like structures or that the spike-like structures are formed by a protein transported across the outer membrane along with the extension of the pilus. Interestingly, in addition to disappearance of the spike-like densities, the *pilE* mutant also showed a 19-fold symmetry similar to the *pilP* deletion mutant and the secretin of wild-type *N. meningitidis*. Phase variation could have changed the expression levels of PilE in the *pilP* mutant and in the *N. meningitidis* strains, and lowered levels of PilE in the *pilE* and *pilP* mutants of *N. gonorrhoeae* and in the *N. meningitidis* strains might explain the change in symmetry. To test this, the expression levels of PilE in *N. gonorrhoeae* strain MS11 and the *pilQ*, *pilP* and *pilE* deletion mutants and in the *N. meningitides* strain HB1, H44/76 and M986 were determined by Western blotting using a PilE-SM1 monoclonal antibody [54] (Figure S4). This demonstrated that PilE expression could be detected in all strains except in the *N. gonorhoeae pilE* deletion mutant and the *N. meningitidis* strain M986. The *N. meningitidis* strain M986 strain expresses a class II pilin that can not be detected with the class I pilE-SM1 antibody [55] but most likely also expresses PilE. Since the *pilP* mutant that has an 19 fold symmetry of the outer ring still expresses PilE, phase variation of PilE expression could be excluded as a possible reason for the structural change of the outer ring of the secretin complex. Why the absence of PilP/PilE in the structure of the *N. gonorrhoeae* PilQ complex results in a structural change to a complex resembling the PilQ complex in *N. meningitidis* remains an open question.

To examine the possible effect of the deletion of a minor pilin, a deletion mutant was created of *pilW*, a minor pilin located in an operon with *pilV* and *pilX*. Single particle analysis was performed and yielded projection maps showing a similar structure as observed for wild-type (Figure 5K and 5L), demonstrating that at least deletion of the *pilW* minor pilin has no effect on the domain structure of the secretin complex.

## Discussion

In this study we analyzed the structure of the PilQ secretin within isolated outer membranes using transmission electron microscopy and single particle averaging. Several lines of evidence demonstrate that the observed stain filled indentations are formed by PilQ: I) The structures are not observed in the *pilQ* deletion mutant (Figure 1A and 1B). II) The structures are also not observed in the *ngonM_03101* mutant. NgonM_03101 is a homologue of *N. meningitidis* PilW. Deletion of the *N. meningitidis pilW gene* was shown to abolish the formation of the PilQ oligomer [56]. III) The symmetry of the outer ring of the structure is affected in deletion mutants of several other *pil* genes, e.g. *pilE*, *pilP*, and *pilF*. It is unlikely that these mutants would affect the structures of another membrane complex than PilQ. IV) The structures are labeled using immuno-gold labeling with an antibody specific for *N. gonorrhoeae* PilQ (Figure 1C). V) The inner ring of strain filled indentations has the same diameter as the purified PilQ complex of *N. meningitidis.* VI) The observed structure is present only in outer membrane sheets. Inner and outer membranes can be easily distinguished in electron microscopy, and the structures are also seen on electron microcopy images of whole cells. VII) The abundance of the structure correlates with the abundance of PilQ in the outer membrane [26]. Our approach revealed features of a large structure not seen previously. Compared to the published structures derived from purified PilQ of *N. meningidis*, the complexes observed in *N. meningitidis* membranes contained an extra peripheral proteinous ring with a 19-fold symmetry. In our analysis, we also observed structures lacking or having a partial peripheral ring, indicating that the extra domains are not tightly attached and may be dissociated during the membrane isolation procedure. Apparently, this extra ring structure is also lost during the previously described purification of the PilQ complex [36]. Also for other purified secretins, no additional ring structures were observed. Only after purification of PulD-PulS complex from *K. oxytoca* radial spokes, most likely formed by PulS were observed [31]. These spokes seem however of a much small mass then the extra ring structure observed for the PilQ complex.

Remarkably, the secretin complexes observed in membranes isolated from *N. gonorrhoeae* appear much more stable, and showed a double ring structure with a 14-fold symmetry of the peripheral ring, from which seven external spikes protrude. These data demonstrate that the study of these multi-component membrane inserted complexes within their native lipid environment by electron microscopy can identify extra components and/or structures which are lost during purification. Compared to the previously published structures derived from purified PilQ complexes of *N. meningitidis*, the central ring in our structures consists of PilQ. The symmetry of the central ring of *N. meningitidis* has previously been determined to be 12, while the symmetries of the *K. oxocyta* PulD and the pIV protein of filamentous phages were 12 and 14, respectively. Unfortunately, we were unable to conclusively determine the symmetry of the central ring of *N. gonorrhoeae*, but our analysis indicates that it is most likely 14, and thus could differ from the central ring of *N. meningitidis*.

Another interesting feature of the secretin complexes investigated is the high flexibility between the different rings and the spikes. In particular, the observation that the number of protein copies in the second ring changes from 14 to 19 in the *pilP* and *pilE* mutants is intriguing. A comparison of the *pilP* and *pilE* mutants with an 19-fold symmetry to those of wild-type and the *pilC1/C2* mutant with an 14-fold symmetry shows that the overall diameter of the peripheral ring is smaller in the *pilP* and *pilE* deletion mutants, whereas the size of the central ring is equal for all complexes. This indicates that it is unlikely that there is a higher copy number of the same protein in the structure with the 19-fold symmetry (which would increase the size of the peripheral ring), but instead suggests that the structure with the 19-fold symmetry either arises from processing of the peripheral ring protein(s), or that the peripheral ring protein(s) are replaced by other protein(s). It appears that the spikes can only attach to the structure with the 14-fold symmetry. Since also structure with a 14-fold symmetry without the spikes are observed it is unlikely that that the presence of spikes forces the inner ring into the 14 fold symmetry.

A comparable change in symmetry between rings has been observed for photosystem I (PSI) of *Synechocystis* PCC. Monomeric photosystem I (PSI) is a membrane protein complex of 330 kDa which is mainly present as trimers in cyanobacteria. Under stress conditions, it forms supercomplexes IsiA, with a 37 kDa integral membrane protein. These complexes have been extensively studied by electron microscopy [57], [58] and it was shown that IsiA can form complete and incomplete single and double rings around monomeric or trimeric PSI. The number of IsiA copies was variable; in the case of monomers the inner IsiA ring was composed of 12, 13 or 14 copies and these numbers corresponded to 19, 20 or 21 copies in the peripheral ring, respectively. On trimers with two IsiA rings, the inner ring is composed of 18 copies and the peripheral ring is formed by 25. However, the positions of IsiA in incomplete second rings with 12–19 copies were slightly different. If extrapolated to the complete rings, they appeared to consist of only 24 copies. These data illustrate how IsiA is flexibly attached to PSI ([57] and unpublished data). Similarly, it is possible that the protein(s) making the second ring around the secretin of the type IV pilus are flexible in their self-association.

Within this study we also attempted to identify the proteins located within the second ring and in the spike-like extensions. Initially, we expected that the peripheral rings and/or spikes were formed by PilC, since PilC is a large (110 kDa) protein which was shown to be located in the outer membrane and at the tip of the pilus [43], [49], and Nme *pilQ* mutants were shown to shed Nme PilC to the medium [52]. However, a mutant of *pilC1/C2* showed similar complexes as observed in wild-type membranes demonstrating that PilC is not a component of the peripheral ring or the spike-like extensions. Another candidate was the homologue of *N. menigitidis* PilW (NgonM_03101), a small (28 kDa) putative lipoprotein containing six tetratricopeptide repeats (TPR) motifs, necessary for the stabilization of pili fibers but not for their assembly or surface localization. A deletion mutant of the *N. gonorrhoeae* homologue of NMe PilW strongly affected the stability of Nme PilQ multimers [41]. Similar results were obtained for the *N. meningitidis* PilW homologues of *Myxococcus xanthus (*Tgl) [59] and of *Pseudomonas aeruginosa* (PilF) [50]. In membranes of the deletion mutant of the *N. gonorrhoeae* homologue of *N. menigitidis* PilW, no secretin complexes are observed, confirming that also the *N. gonorrhoeae* NgonM_03101 affects the stability of PilQ. Therefore we cannot rule out that NgonM_03101 is part of the second ring or the spikes, although the small size of the protein might not account for the densities of the peripheral ring subunits.

Interestingly, our study demonstrated that the symmetry of the peripheral ring of the secretin complex in the *pilP* and *pilE* deletion mutants changed from 14 to 19, and that the structure lost the extending spike-like densities in the *pilP*, *pilE* and *pilF* deletion mutants. These results demonstrate that both PilE and PilP are important for the assembly of the peripheral ring. PilP is a small protein (21 kDa) previously suggested to be localized in the inner membrane, and to attach to the cap region of the PilQ complex [36]. This would place the PilP protein on the periplamic interface possibly between the central and peripheral ring. These data and the small size of PilP make it unlikely that PilP forms either the second ring or the spike-like extension, but PilP could be involved in aligning the central and peripheral ring. The effects of mutations in the pilin protein PilE, and the PilF secretion ATPase, which both inhibit formation of the pilus structure, demonstrate that pilus formation influences the PilQ complex. The changes observed in the PilQ complexes can be a direct effect of an interaction between the pilus and PilQ, or an indirect effect on the export or assembly of minor pilins or pilus associated proteins in the absence of a formed pilus. Our data cannot discriminate between these two possibilities.

Our approach has revealed that the PilQ secretin complex of the type IV pili of *Neissera gonorrhoeae* interacts with other proteins in the peripheral membrane to form a large multi-domain complex. The function of these extra domains is currently unknown, but they may simply be involved in anchoring the secretin stably into the outer membrane during pilus extension and retraction. Alternatively, the extra domains could be involved in attaching proteins to the pilus, modifying the pilus or play a specific role in type IV pili dependent natural transformation. It will be important to identify the protein within the extra domains and to determine whether these domains can also be found in Type II secretion systems or in the Type IV pili systems of other organism.

## Materials and Methods

### Strains, plasmids, primers and media

Strains used in this study are described in Table 1. *N. gonorrhoeae* strains were grown at 37°C in 5% CO_2_ on GCB (Difco) plates containing Kellog's supplement [60] or GCB liquid medium (GCBL) containing 0.042% NaHCO_3_ and Kellog's supplements. *N. meningitidis* was also grown at 37°C in 5% CO_2_ on GC-agar plates or in tryptic soy broth (TSB). When necessary, erythromycin was used at 5 µg/ml.

**Table 1:** Strains used in this study.

Strains	Description	Reference
HB1	*Neisseria meningitidis* strain	[68]
M986	*Neisseria meningitidis* strain	[69]
MS11	*Neisseria gonorrhoeae* strain	[6]
FA1090	*Neisseria gonorrhoeae* strain	[70]
SJ001-MS	MS11 strain with *pilQ* truncation	This work
SJ030-MS	MS11 strain transformed with pSJ030; non polar insertion in *pilC*, ErmC	This work
SJ031-MS	MS11 strain transformed with PCR product to introduce in frame deletion of aa 1 to 181 of *pilP*	This work
SJ007-MS	MS11 strain transformed with PCR product to introduce in frame deletion of aa 1 to 145 of *NgonM_03101*	This work
EP060	MS11 strain transformed with pEP057; non polar Insertion in *pilF*, ErmC	This work
SJ006-FA1090	FA1090 strain transformed with PCR product to introduce in frame deletion of aa 1 to 31 of *pilE*	This work
SJ002-MS	MS11 transformed with SBR repeat containing *pilQ* region of HB1	This work
SJ032-MS	MS11 strain transformed with pSJ032; non polar insertion of *pilW*, ErmC	This work

### Construction of deletion mutant strains

Deletion mutants in *pilC* and *pilF* were made using the insertion-duplication mutagenesis method [61]. Using this method, the gene is disrupted and expression of genes downstream of the disrupted gene is driven from the erythromycin promotor. PCR products encoding 541 bp (primers PilC-for and PilC-rev), 524 bp (primers PilF-for and PilF-rev) and 452 bp (primers PilW-for and PilW-rev) fragments of *pilC*, *pilF* and *pilW* were amplified from isolated chromosomal DNA of *N. gonorrhoeae* strain MS11 (for a list of used primers, see Table 2). The *pilC* and *pilW* PCR fragment was digested with *BamH*I and *Kpn*I and ligated into the *BamH*I/*Kpn*I sites of pIND3 [62], resulting in plasmid pSJ030 and pSJ032 respectively. The *pilF* PCR fragment was digested with *Xho*I and *Kpn*I and ligated into *Xho*I/*Kpn*I site of pIND3, resulting in plasmid pEP057. Plasmids pSJ030, pSJ032 and pEP057 were transformed to MS11 and colonies were selected on GCB plates containing erythromycin. Correct clones were identified by performing a PCR on isolated chromosomal DNA of these colonies resulting in strains SJ030-MS, SJ032-MS and EP060, respectively (Table 1). To create marker-less non-polar deletion mutants of *pilP*, *NgonM_03101*, *pilQ* and *pilE*, PCR fragments of the flanking regions of the respective genes were annealed using the splicing by overlapping extension PCR (SOE-PCR) [63] method. To create the PCR products for *pilP*, *NgonM_03101*, *pilQ* and *pilE*, the primer combinations of PilP-for1/PilP-rev1 and PilP-for2/PilP-rev2; NgonM_03101-for1/NgonM_03101-rev1 and NgonM_03101-for2/NgonM_03101-rev2, PilQ-for1/PilQ-rev1 and PilQ-for2/PilQ-rev2 and PilE-for1/PilE-rev1 and PilE-for2/PilE-rev2 were used. The obtained PCR products were diluted and amplified with the external primers which also contained the gonoccocal DNA uptake sequence (DUS). The PCR product was transformed to strain MS11 or FA1090 and the mutant colonies were checked using colony PCR. The marker-less insertion of the SBR containing region of the *N. meningitidis* HB1 strain was introduced into *N. gonorrhoeae* MS11 by transformation of a PCR fragment carrying the extra region. The PCR product was obtained by using the SBR-for and SBR-rev primers. Correct clones were identified by performing a PCR on isolated chromosomal DNA of several colonies resulting in strains SJ031-MS, SJ007-MS, SJ001-MS, SJ006-FA1090 and SJ002-MS (Table 1). To further confirm the correct deletion of the gene, the deletion site and the flanking regions were determined by sequencing.

**Table 2:** Primers used in this study.

PilC-for	5′-TGGCGGTACCCTCGCTGCCCAAATTGAAAG-3′
PilC-rev	5′-GCGCGGATCCGTAAATACGCTATTATCATGGACG-3′
NgonM_03101-for1	5′-GACGTCATAGTCAGCAATTACCCCTGTTGTCCGATTAAA-3′
NgonM_03101-rev1	5′-TTCAGACGGCATGACCACGGCTACGGTTTGAG-3′
NgonM_03101-for2	5′-ATGCCGTCTGAAGCTGTTGACGGTAATCCGCAC-3′
NgonM_03101-rev2	5′-GCTGACTATGACGTCCCTGAATAAAGGTATATGCAGCG-3′
PilF-for	5′-ATGCTCGAGACGGCGCGACACCCATATTC-3′
PilF-rev	5′-TACGGTACCCGGCAAGCCTGTCGATTTCC-3′
PilP-for1	5′-GGTTTCCCTAACGTAAGTTATTTTTGCTCGGCATTTTGTG-3′
PilP-rev1	5′-ATGCCGTCTGAACAACCTATCGTAAAGGCGGCCGAATCCAA-3′
PilP-for2	5′-TTCAGACGGCATGCCGCCAATTCGATAATGCC-3′
PilP-rev2	5′-ACTTACGTTAGGGAAACCATGAATACCAAACTGACAAAAATC-3′
PilQ-for1	5′-GCTGACTATGACGTCCAGACATCAAAGTTTCCTCCCTGCC-3′
PilQ-rev1	5′-TTCAGACGGCATGCCGCCAATTCGATAATGCC-3′
PilQ-for2	5′-ATGCCGTCTGAAACCTTTCCAAGCACCTACCC-3′
PilQ-rev2	5′-GACGTCATAGTCAGCATGGAGTAATCCTCTTCTTAAT-3′
PilE-for1	5′-ATGCCGTCTGAAGCCTTATTTGGCAGTTGG
PilE-rev1	5′-GCTGACTATGACGTCCCTACCAAGACTACACCGCCC
PilE-for2	5′-TTCAGACGGCATACGCTTCATCTGCCGGTTGCATAG
PilE-rev2	5′-GACGTCATAGTCAGCGGCGACAACTGCGTATTATAAAGC
SBR-for	5′-GCTGACTATGACGTCCAGACATCAAAGTTTCCTCCCTGCC-3′
SBR-rev	5′-TTCAGACGGCATGCTTTGTCTTTGGCAAGCAG-3′
PilW-for	5′-GCGCGGTACCTTGTCCGCGATGCAAGAATG-3′
PilW-rev	5′-GCGCGGATCCCGACCGCATAGGCATTGACCAC-3′

### Membrane Preparation

To isolate membranes of *N. gonorrhoeae*, the strain was plated on GCB plates with the appropriate antibiotic, and (when possible) piliated cells were scraped from the plate and transferred to 3 ml GCBL medium. Cells were grown to an OD_660_ of 0.6 and consecutively diluted into increasing volumes until a final volume of 1 liter with an OD_660_ of 1.0 was obtained. Cells were centrifuged at 8,000 rpm in a JLA-16.25 rotor and resuspended in 50 mM Tris-HCl pH 7.5. Cells were broken by three passes through a French press at 15 kpsi. Cell debris was removed by centrifugation at 6,000 rpm in a SS34 rotor for 10 min. The membranes were pelleted at 40,000 rpm in a Ti-45 rotor for 1 h, resuspended in 1 ml of 50 mM Tris-HCl pH 7.5 and overlaid on a 4 step (1, 1.8, 0.8 and 0.8 ml) sucrose gradient of 54, 51, 45 and 36 (w/v) sucrose) and centrifuged at 80,000 rpm in a MLA-80 rotor for 30 min. The lower two fractions were collected, diluted in 50 mM Tris-HCl pH 7.5, membranes were collected by centrifugation at 40,000 rpm in a Ti-45 rotor for 1 h, and resuspended in 1 ml 50 mM Tris-HCl pH 7.5. To isolate membranes of *N. meningitidis*, the strain was inoculated from an overnight GC-agar plate in 40 ml tryptic soy broth at an OD_550_ of 0.1 and grown for 7 h to OD_550_ of 4. Cells were collected by centrifugation, resuspended in 50 mM Tris/HCl, 5 mM EDTA pH 8 and frozen at −80°C. After thawing, aliquots were plated to verify killing of the bacteria. The suspensions were sonicated for 4 min (Branson 450, setting 6, output 40%) and spun at 10,000 rpm in an SS34 rotor. The resulting supernatant was spun for 8 min at 40,000 rpm in a Ti-70. Cell envelope pellets were dissolved in 2 mM Tris/HCl pH 7.6 and overlaid on a 4 step (1, 1.8, 0.8 and 0.8 ml) sucrose gradient of 54, 51, 45 and 36% (w/v) sucrose and centrifuged at 80,000 rpm in a MLA-80 rotor for 30 min. The lower two fractions were collected, diluted in 50 mM Tris-HCl pH 7.5 and membranes were collected by centrifugation at 40,000 rpm in a Ti-45 rotor for 1 h. The final membrane preparation was resuspended in 50 mM Tris-HCl, pH 7.5, and used for EM analysis.

### Electron Microscopy and single particle analysis

For image processing, whole membranes from *N. gonorrhoeae* and *N. meningitidis* were negatively stained with 2% uranyl acetate on glow-discharged carbon-coated copper grids. Electron microscopy was performed on a Philips CM120 equipped with a LaB6 tip operating at 120 kV. The “GRACE” system for semi-automated specimen selection and data acquisition [64] was used to record 2048×2048 pixel images at 60,000x calibrated magnification with a Gatan 4000 SP 4K slow-scan CCD camera. About 9,000 images were recorded.

From the images we selected about 20,000 single particle projections of the PilQ complex from *N. gonorrhoea*, 8,000 projections of the PilQ complex from *N. meningitidis*, 7,000 projections of the *pilC* deletion mutant and approximately 5,000 of the *pilE* and *pilP* deletion mutants from *N. gonorrhoea*, respectively. Single particle analysis was performed using the Groningen Image Processing (“GRIP”) software packages (see http://bfcemw0.chem.rug.nl/progs-grip.html for a description) on PC clusters. Single particles of PilQ were repeatedly aligned with multireference and nonreference alignments and treated with multivariate statistical analysis and hierarchical ascendant classification [65]. In the final step, the best 50% of the class-members of the best 50% of the classes were taken for the final sums, with the correlation coefficient from alignments as a quality parameter. Rotational symmetry was analyzed in a similar way, as described previously [66].

### Nanogold labeling of isolated membranes with PilQ antibodies

5 µl of the outer membrane enriched fraction of wild type *N.gonorrhoea* MS11A strain was immobilized on a glow-discharged carbon-coated copper grid. The grid was then incubated with *N.meningitidis* PilQ monoclonal antibody [46] diluted 1∶1 in wash buffer (20 mM Tris-HCl pH 7.5, 150 mM NaCl) for 1 hr. After 3 washes with wash buffer, the grid was incubated for 1 hr with 1∶10 diluted gold labeled Protein G secondary antibody (Aurion, The Netherlands). After 3 washes with wash buffer the sample was fixed with 2% glutaraldehyde for 5 minutes before staining with 2% uranyl acetate. Electron microscopy was then performed as described above. To exclude aspecific labeling, membranes were labeled using a similar protocol as described above, with the difference that the PilQ monoclonal antibody was replaced by buffer. To test whether the labeling was specific for the presence of the indentations, membranes from the outer membrane enriched fractions of the wild type *N.gonorrhoea* MS11A strain and the *pilQ* deletion mutant strain were mixed and immobilized on a grid. Labeling was then performed as above.

### Electron microscopy on whole cells

Piliated and non-piliated colonies of *N. gonorrhoeae* strain MS11 were selected and transfered to GCB plates. After 18 hrs of growth, the cells were scraped from the surface of the plate and resuspended in 1 ml of GCBL medium. 5 µl of this suspension was incubated on a glow-discharged carbon-coated copper grid. Carbon grids were then washed three times with water before straining with uranyl acetate. The grids were analyzed by electron microscopy as described above.

### SDS-PAGE and Western blotting

In order to test the cross-reactivity of the PilQ monoclonal antibody [46] directed against *N.meninigitidis* PilQ to that of *N.gonorrhoeae* PilQ; isolated outer membrane enriched samples were treated with phenol to generate momomeric PilQ as described previously [67]. Briefly, 200 µl (about 500 µg protein) was mixed with equal volume of 88% phenol and incubated at 70°C for 10 minutes. The samples were then cooled to 4°C and centrifuged at 5000× g for 10 minutes. The upper aqueous phase was discarded and the intermediate and lower phase was retained and mixed with equal volume of water. After incubation at 70°C for 10 minutes, samples were centrifuged at 5000× g for 10 minutes to remove the aqueous phase once again. The protein was then precipitated with 1 ml ice cold acetone and the pellet was resuspended in sample buffer and run on a 10% SDS-PAGE gel either for coomassie staining or for immunoblotting.

Western blotting was performed using PVDF membranes. Blots were developed by incubating with a 1∶1000 dilution of the PilQ [46] and the PilE monoclonal [54] antibody, followed by washes, and incubation with a 1.10000 dilution of anti-Mouse alkaline phosphatase-conjugated secondary antibody (Sigma). The chemiluminescence signal was obtained using the CSDP-star substrate (Roche) on a Roche Lumi-imager.

## Supporting Information

Figure S1 **PilQ is a major outer membrane protein in Neisseria species.** (A) Coomassie stained PAGE gel and (B) Western blot using the monoclonal antibody raised against *N.meningitidis* PilQ [1] of phenol treated outer membrane enriched samples from *N.meningitidis* (lane 1 and 4), *N.gonorrhoeae* MS11 (lane 2 and 5) and the *N.gonorrhoeae pilQ* mutant (lane 3 and 6).
(DOCX)

Figure S2 **Nanogold labeling of isolated** ***N.gonorrhoeae*** **membranes with PilQ monoclonal antibody.** Membranes are labeled with PilQ antibody-gold conjugate. Besides uncoated pores (red boxes), some pores are covered with gold clusters; the fact that some of the clusters are right on top of the pores is visible from the bright circular circumference or “halo” around the gold clusters (green boxes). The scale bar is 100 nm.
(DOCX)

Figure S3 **The PilQ monoclonal antibody specifically labels PilQ.** (A) Labeling of membranes of *N.gonorrhoeae* MS11 (WT) with secondary antibody-gold conjugate in the absence of the primary PilQ antibody shows no gold-conjugates. (B) Labeling of mixed membranes of the *N.gonorrhoeae pilQ* mutant (left) and *N.gonorrhoeae* MS11 (WT, right) on the same electron microscopy grid with PilQ antibody, followed by detection with a secondary antibody-gold conjugate directed against the PilQ antibody labels only membranes containing PilQ (right) Both pictures are representative selections of the same grid.
(DOCX)

Figure S4 **Immunoblots on Neisseria membranes with PilE and PilQ antibody.** (A) Western blot using a monoclonal antibody raised against *N.meningitidis* PilE-SM1. Lanes show outer membrane enriched samples from *N.gonorrhoeae* MS11 (lane 1), and the *pilQ* (Lane 2), *pilP* (Lane 3) and *pilE* mutants (Lane 4), and *N.meningitidis* strains M986 (lane 5), H44/76 (lane 6) and HB1 (lane 7). (B) Western blot using the monoclonal antibody raised against *N.meningitidis* PilQ on phenol treated outer membrane enriched samples from *N.gonorrhoeae* MS11 (lane 1), and *pilQ* (Lane 2), *pilP* (Lane 3), and *pilE* (Lane 4) mutants.
(DOCX)
