# Supplementary material for: Structural Characterization of Outer Membrane Components of the Type IV Pili System in Pathogenic Neisseria
Source: PLoS One. 2011 Jan 31;6(1):e16624. doi: 10.1371/journal.pone.0016624 (PMC3031610; doi:10.1371/journal.pone.0016624)
Supplement: Figure S1 — PilQ is a major outer membrane protein in Neisseria species. (A) Coomassie stained PAGE gel and (B) Western blot using the monoclonal antibody raised against N.meningitidis PilQ [1] of phenol treated outer membrane enriched samples from N.meningitidis (lane 1 and 4), N.gonorrhoeae MS11 (lane 2 and 5) and the N.gonorrhoeae pilQ mutant (lane 3 and 6). (DOCX) [file pone.0016624.s001.docx]

**Supporting Information Jain *et al.***

**Figure S1**

**
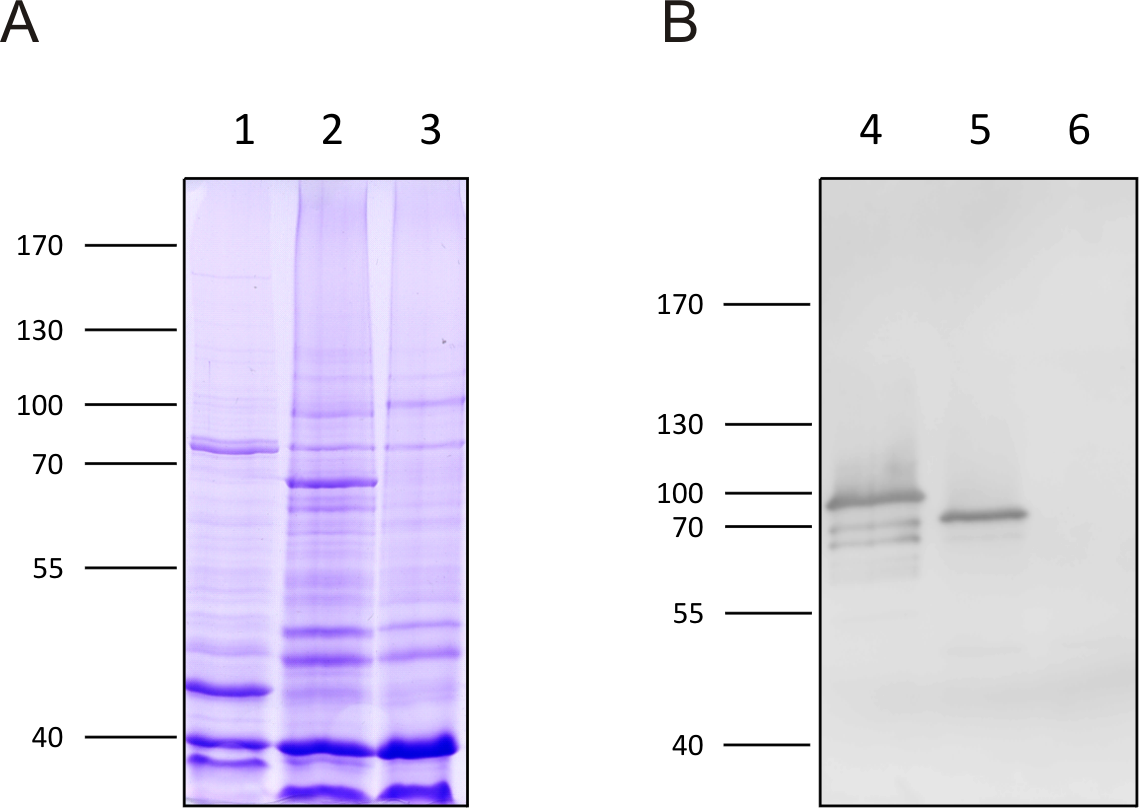
**

**Figure S1. PilQ is a major outer membrane protein in Neisseria species.** (A) Coomassie stained PAGE gel and (B) Western blot using the monoclonal antibody raised against *N.meningitidis* PilQ [[1](#_ENREF_1)] of phenol treated outer membrane enriched samples from *N.meningitidis* (lane 1 and 4), *N.gonorrhoeae* MS11 (lane 2 and 5) and the *N.gonorrhoeae* *pilQ* mutant (lane 3 and 6 ).
