# Supplementary material for: Structural Characterization of Outer Membrane Components of the Type IV Pili System in Pathogenic Neisseria
Source: PLoS One. 2011 Jan 31;6(1):e16624. doi: 10.1371/journal.pone.0016624 (PMC3031610; doi:10.1371/journal.pone.0016624)
Supplement: Figure S2 — Nanogold labeling of isolated N.gonorrhoeae membranes with PilQ monoclonal antibody. Membranes are labeled with PilQ antibody-gold conjugate. Besides uncoated pores (red boxes), some pores are covered with gold clusters; the fact that some of the clusters are right on top of the pores is visible from the bright circular circumference or “halo” around the gold clusters (green boxes). The scale bar is 100 nm. (DOCX) [file pone.0016624.s002.docx]

**Supporting Information Jain *et al.***

**Figure S2**


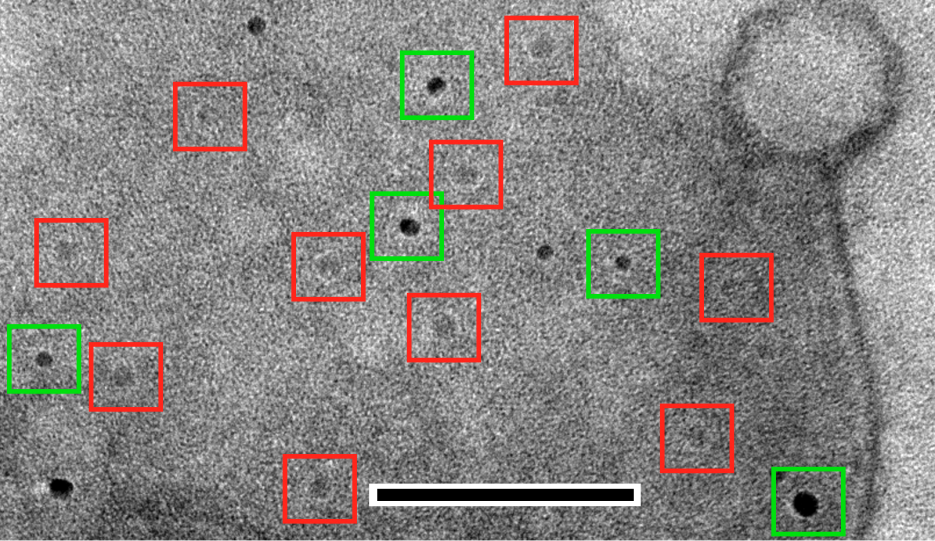


**Figure S2. Nanogold labeling of isolated *N.gonorrhoeae* membranes with PilQ monoclonal antibody*.*** Membranes are labeled with PilQ antibody-gold conjugate. Besides uncoated pores (red boxes), some pores are covered with gold clusters; the fact that some of the clusters are right on top of the pores is visible from the bright circular circumference or “halo” around the gold clusters (green boxes). The scale bar is 100 nm.
