# Supplementary material for: Structural Characterization of Outer Membrane Components of the Type IV Pili System in Pathogenic Neisseria
Source: PLoS One. 2011 Jan 31;6(1):e16624. doi: 10.1371/journal.pone.0016624 (PMC3031610; doi:10.1371/journal.pone.0016624)
Supplement: Figure S3 — The PilQ monoclonal antibody specifically labels PilQ. (A) Labeling of membranes of N.gonorrhoeae MS11 (WT) with secondary antibody-gold conjugate in the absence of the primary PilQ antibody shows no gold-conjugates. (B) Labeling of mixed membranes of the N.gonorrhoeae pilQ mutant (left) and N.gonorrhoeae MS11 (WT, right) on the same electron microscopy grid with PilQ antibody, followed by detection with a secondary antibody-gold conjugate directed against the PilQ antibody labels only membranes containing PilQ (right) Both pictures are representative selections of the same grid. (DOCX) [file pone.0016624.s003.docx]

**Supporting Information Jain *et al.***

**Figure S3 A**

**
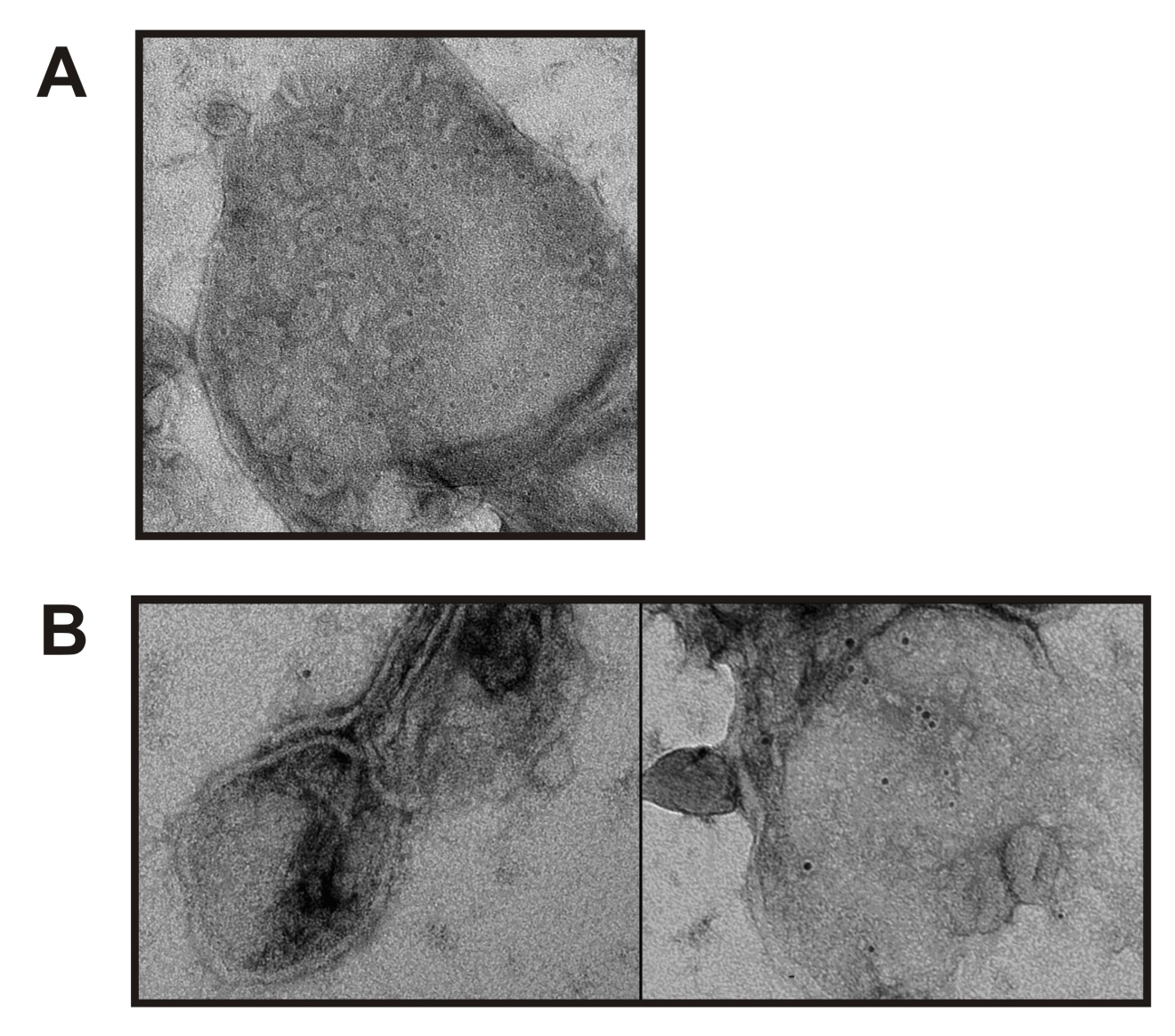
**

**Figure S3. The PilQ monoclonal antibody specifically labels PilQ**

(A) Labeling of membranes of *N.gonorrhoeae* MS11 (WT) with secondary antibody-gold conjugate in the absence of the primary PilQ antibody shows no gold-conjugates. (B) Labeling of mixed membranes of the *N.gonorrhoeae* *pilQ* mutant (left) and *N.gonorrhoeae* MS11 (WT, right) on the same electron microscopy grid with PilQ antibody, followed by detection with a secondary antibody-gold conjugate directed against the PilQ antibody labels only membranes containing PilQ (right) Both pictures are representative selections of the same grid.
