# Supplementary material for: Structural Characterization of Outer Membrane Components of the Type IV Pili System in Pathogenic Neisseria
Source: PLoS One. 2011 Jan 31;6(1):e16624. doi: 10.1371/journal.pone.0016624 (PMC3031610; doi:10.1371/journal.pone.0016624)
Supplement: Figure S4 — Immunoblots on Neisseria membranes with PilE and PilQ antibody. (A) Western blot using a monoclonal antibody raised against N.meningitidis PilE-SM1. Lanes show outer membrane enriched samples from N.gonorrhoeae MS11 (lane 1), and the pilQ (Lane 2), pilP (Lane 3) and pilE mutants (Lane 4), and N.meningitidis strains M986 (lane 5), H44/76 (lane 6) and HB1 (lane 7). (B) Western blot using the monoclonal antibody raised against N.meningitidis PilQ on phenol treated outer membrane enriched samples from N.gonorrhoeae MS11 (lane 1), and pilQ (Lane 2), pilP (Lane 3), and pilE (Lane 4) mutants. (DOCX) [file pone.0016624.s004.docx]

**Supporting Information Jain *et al.***

**Figure S4**

**
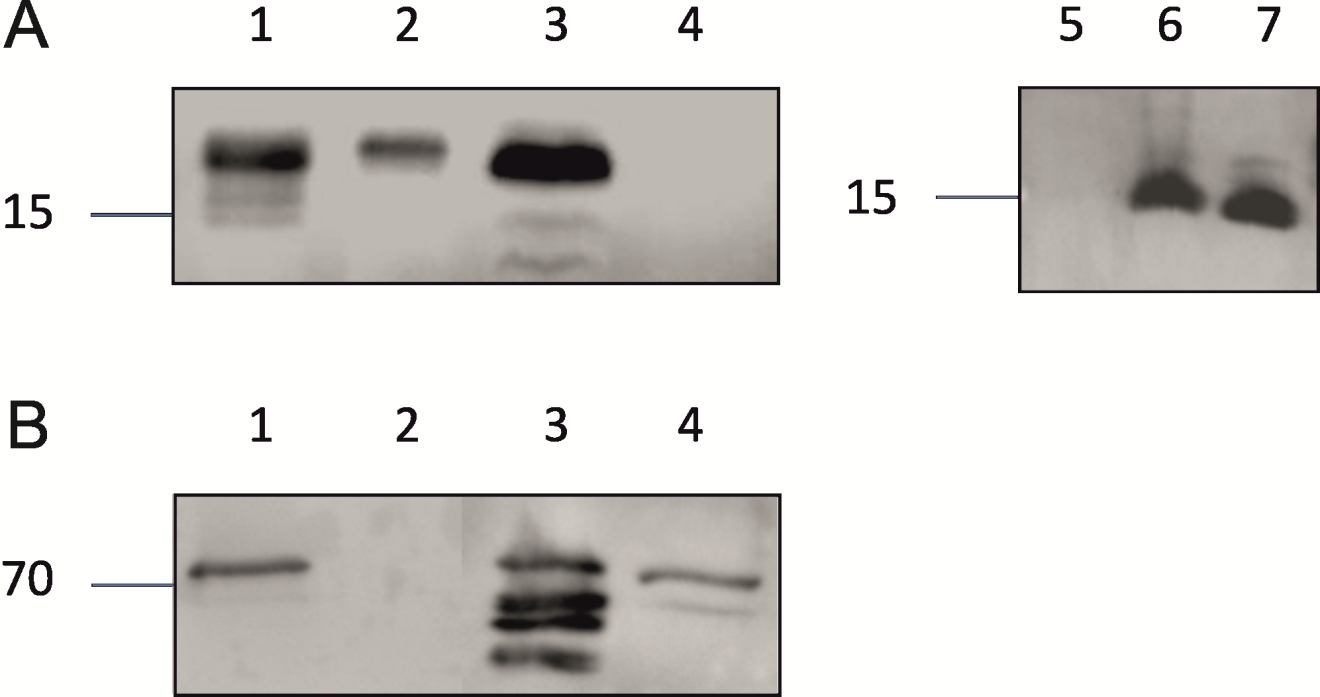
**

**Figure S4. Immunoblots on Neisseria membranes with PilE and PilQ antibody.** (A) Western blot using a monoclonal antibody raised against *N.meningitidis* PilE-SM1. Lanes show outer membrane enriched samples from *N.gonorrhoeae* MS11 (lane 1), and the *pilQ* (Lane 2), *pilP* (Lane 3) and *pilE* mutants (Lane 4), and *N.meningitidis* strains M986 (lane 5), H44/76 (lane 6) and HB1 (lane 7). (B) Western blot using the monoclonal antibody raised against *N.meningitidis* PilQ on phenol treated outer membrane enriched samples from *N.gonorrhoeae* MS11 (lane 1), and *pilQ* (Lane 2), *pilP* (Lane 3), and *pilE* (Lane 4) mutants.
